# Impact of Helminth Infections and Nutritional Constraints on the Small Intestine Microbiota

**DOI:** 10.1371/journal.pone.0159770

**Published:** 2016-07-20

**Authors:** Isabella M. Cattadori, Aswathy Sebastian, Han Hao, Robab Katani, Istvan Albert, Kirsten E. Eilertson, Vivek Kapur, Ashutosh Pathak, Susan Mitchell

**Affiliations:** 1 Center for Infectious Disease Dynamics, The Pennsylvania State University, University Park, 16082 PA, United States of America; 2 Department of Biology, The Pennsylvania State University, University Park, 16082 PA, United States of America; 3 Department of Biochemistry and Molecular Biology, The Pennsylvania State University, University Park, 16082 PA, United States of America; 4 Department of Statistics, The Pennsylvania State University, University Park, 16082 PA, United States of America; 5 Department of Veterinary and Biomedical Sciences, The Pennsylvania State University, University Park, 16082 PA, United States of America; Universidade de Aveiro, PORTUGAL

## Abstract

Helminth infections and nutrition can independently alter the composition and abundance of the gastrointestinal microbiota, however, their combined effect is poorly understood. Here, we used the *T*. *retortaeformis*-rabbit system to examine how the helminth infection and host restriction from coprophagy/ready-to-absorb nutrients affected the duodenal microbiota, and how these changes related to the acquired immune response at the site of infection. A factorial experiment was performed where the bacterial community, its functionality and the immune response were examined in four treatments (Infect, Infect+Collar, Control+Collar and Control). Helminths reduced the diversity and abundance of the microbiota while the combination of parasites and coprophagic restriction led to a more diversified and abundant microbiota than infected cases, without significantly affecting the intensity of infection. Animals restricted from coprophagy and free from parasites exhibited the richest and most abundant bacterial community. By forcing the individuals to absorb nutrients from less digested food, the coprophagic restriction appears to have facilitated the diversity and proliferation of bacteria in the duodenum. Changes in the microbiota were more clearly associated with changes in the immune response for the infected than the nutrient restricted animals. The functional and metabolic characteristics of the duodenal microbiota were not significantly different between treatments. Overall, infection and diet affect the gut microbiota but their interactions and outcome can be complex. These findings can have important implications for the development of control measures to helminth infections where poor nutrition/malnutrition can also be a concern.

## Introduction

The commensal microbiota of the gastrointestinal tract is a dynamic ecosystem that has to adjust to the repeated disturbance exerted by external factors while maintaining the homeostasis and functionality of the individual [1–4]. Infectious diseases and dietary changes are important sources of disturbance for the immune system and there is increasing evidence that they can also alter the composition, abundance and functionality of the bacterial community in the digestive tract [5–10]. Individuals exposed to endemic helminth infections have been shown to have increased microbiota richness and enhanced functional activities such as genetic processing, metabolism and cell cycling, compared to non-infected cases from developing and industrialized countries [11]. Well characterized also is the imbalance of the gut microbiota in obese and malnourished groups [5, 12, 13] and the significant diet-related diversity between the bacterial community of modern culture and hunter/gatherer societies [14, 15]. However, while the role of the gut microbiota on host health is now well recognized, how the microbiota is affected by helminth infections and nutritional alterations, and how these interactions relate to host immunity, remains poorly understood. Given that approximately a quarter of the world population is infected with soil-transmitted helminths [16] that cause persistent, often subclinical disease and no life-long immune protection, and considering that parasites can also impact host metabolism and nutrient absorption [17, 18], understanding the interaction between helminths and the gut microbiota is essential for developing new preventive approaches that can promote gastrointestinal health and overall nutrition.

Coprophagy is a feature commonly observed in many mammal species [19–21] and the therapy of fecal transplantation for the re-establishment of the microbiota dysbiosis has many similarities with this behavior [22, 23]. The daily ingestion of feces by-products of the bacterial metabolism in the cecum (cecotrophy: eating cecotropes) is unique to lagomorphs and has been well investigated in rabbits by showing that it is associated with the absorption of fundamental proteins and nitrogen [24–26]. This behavior can be considered a natural mode of nutrient enrichment [27] but also a way of boosting the animal's microbiota in the gastrointestinal tract. Indeed, by ingesting cecal feces it is possible that individuals could maintain a more diversified and functionally balanced microbial community as well as mitigate the severity to local infections, such as gastrointestinal helminths; although parasites have also been suggested to contribute to microbiota enhancement [28, 29].

Helminth infections commonly induce two complementary host immune responses: a type 2 protective response directed at reducing parasite establishment and survival and a regulatory response to mitigate the impact of the worm population on host tissues, for instance, reducing the damage caused by the movements of immature stages into the gut wall during development [30, 31]. Likewise, the gut microbiota has been shown to be under the direct control of an adaptive and regulatory immune response that restrains the bacteria from proliferating or degenerating into pathogenic phenotypes [6, 32]. However, the gastrointestinal microbiota can also contribute to modulating the local tolerigenic and defensive immune responses [6, 33–37]. For instance, *Bacteroides* are more effective in stimulating the production of mucosa secretory IgA than *Lactobacilli* [38], and different species of *Lactobacilli* can regulate dendritic cells (DC) or activate natural killer (NK) cells [39]. Despite these emerging properties, the relationship between the gastrointestinal microbiota and the local immune response during helminth infections is fundamentally unknown (but see [40]). On one hand, helminths could stimulate bacterial diversity, abundance and functional variability and, thus, enhance local tolerance or defense to the helminth infection. On the other hand, by disrupting the microbial structure and functionality, they could suppress the protective and tolerogenic qualities of the bacteria. Nutritional restrictions are expected to alter these interactions, either by depauperating or ameliorating the microbiota, and/or the impact of the parasite on the local homeostasis.

To examine how helminth infections and nutritional constraints alter the host microbiota and how this relates to the immune response, we investigated the effect of *Trichostrongylus retortaeformis* and cecotrophic limitations on the structure and functionality of the small intestinal microbiota of rabbits (*Oryctolagus cuniculus*). Our hypothesis was that rabbits with helminths carried an impoverished bacterial community, and nutritional restrictions, by cecotrophic prevention, were expected to exacerbate this trend. We also anticipated both positive and negative changes in some of the components of the immune response and microbiota functionality proportional to the intensity of infection and the impact of nutritional restrictions, for example, an increase of a type 2 immune reaction in animals with parasites and restricted nutrition. This host-helminth system has many similarities with parasite infections of human and livestock and can provide fundamental knowledge on the interactions between parasitic and commensal species, their changes over the course of the infection and their association with the local immune response.

## Approach and Methods

### The helminth-rabbit system

*Trichostrongylus retortaeformis* colonizes the small intestine, preferentially the duodenum of the rabbit [41, 42]. Once ingested, third stage infective larvae (L3) mature into adults after a brief phase spent in the mucosal tissue; the pre-patent period is about 12 days [41]. In natural settings, the parasite load accumulates with host age, peaks in young and decreases in older animals as the rabbits are able to control the infection [43, 44]. We previously showed that *T*. *retortaeformis* stimulates a type 1—type 2 mucosal immune response in that an initially high IFNγ expression quickly wanes during the course of the infection to values that are comparable to IL4 and IL10 [45]. At the tissue level, we found significant villous atrophy, increased crypt hyperplasia and local recruitment of eosinophils and lymphocytes, together with a rapid production of mucus IgA and IgG to L3 and adult stages [45].

### Experimental design and sampling

Outbred, two months old, New Zealand white male rabbits (Harlan, US) were housed in single cages with a 12h light cycle and daily access to 125g of standard pellet (LabDiet: rabbit HF 5326: carbohydrate-nitrogen free 42.8%, fiber 22.5%, protein 14.8%, lipid 5.8%, minerals 6.8%) and water *ad libitum*. Our factorial experiment was based on 4 treatments sampled at 3 time points. Groups of 12 animals were randomly assigned to the treatments: Control (C, untreated), Infect (I, helminth dosed), Control with Collar (CC, untreated but with Elizabethan collar) and Infect with Collar (IC, helminth dosed with Elizabethan collar) for a total of 48 animals. Four additional rabbits were also used to quantify the baseline initial conditions at day 0 (B, baseline cases). Commercially available, transparent Elizabethan collars (PetSmart Inc., USA) were fitted around the neck of the rabbits one week before the start of the experiment (day 0), to allow the microbiota to adjust to this change and the animals to get used to the collars. Animals adapted to the collars in few hours and showed no evidence of pain or distress throughout the trial. Elizabethan collars are commonly used to prevent oral-nasal contact of the animal with other parts of its body and have been widely used in nutritional studies with rabbits to restrain animals from cecotrophy. At the end of the experiment, we noticed that rabbits with collars could eat cecotrope fragments from the cage floor (i.e. the small remains of feces that passed through the cage holes). While we do not exclude that few animals ingested some of these remains, this did not affect out results and conclusions.

Infected animals were orally gavaged (Centurion MP, Williamston, MI) every 7 days with 1000 *T*. *retortaeformis* L3 suspended in 3 ml of tap water; Control and Control+Collar animals were sham inoculated with tap water. For every treatment, groups of 4 individuals were sampled at days 15, 30 and 60 days post infection; baseline cases were also sampled at day 0. These sampling points were selected to provide important host and parasite information at the time when: 1- the first parasite eggs are shed into the gut lumen (15 days post initial infection), 2- the host immune response is expected to exhibit the strongest type 1 response (30 days post initial infection) and 3- the intensity of infection and the type 2 immune response are representative of a chronic condition (60 days post initial infection). At each sampling point and for every individual, the small intestine was collected and divided into 3 sections, the first section (duodenum) was selected and further divided into 3 segments of equal length that were then used for the microbial, immunological and parasitological work, respectively. This sampling provided a reliable representation of parasite abundance while accounting for possible spatial variability [45, 46]. All the animal procedures were approved by the Institutional Animal Care and Use Committee of The Pennsylvania State University (USA) and carried out in accordance with the approved guidelines. No animals became ill or died prior to the experimental endpoint.

### Microbiome DNA extraction and genomics

The DNA extraction was performed following the protocol from the Mo Bio Powersoil DNA isolation kit (Carlsbad, CA, USA) with minor modifications. A piece of the duodenum (5 cm) was gently washed with PBS to remove the ingesta and then cut longitudinally before collecting the mucosal lining using a sterile swab applicator (Puritan Medical Products, Guilford, Maine). The cotton tip was then cut, placed directly into the tubes containing lysis beads and gently vortex to allow the sample to be dispersed into the solution. Heated lysis buffer (60°C) was added to the tubes, vortex briefly and then placed in a bead beater for 10 minutes at maximum speed for thorough homogenization. Sterile DNA-Free PCR Grade Water (Teknova, Hollister, CA) was used for DNA elution. Experimental negative samples were included for the DNA extraction and amplification steps and were consistently negative. The DNA concentration was quantified with a NanoDrop Spectrophotometer and a Qubit 2.0 Fluorometer (Life Technologies, Carlsbad, CA) following manufacturer's instructions. The results showed that background amplification or sequence contamination were not an issue and hence accounted for. The procedure was performed in duplicate tissue samples from each animal.

Following preliminary 16S rRNA gene sequencing and bioinformatics we selected the region V3V5 as a good candidate for identifying organisms at the species level. From this highly conserved V3V5 region, the primers 347F (GGAGGCAGCAGTRRGGAAT) and 803R (CTACCRGGGTATCTAATCC) were chosen to characterize the microbial community [47–49]. The PCR products (1000 bps) were purified using the AgencourtAMPure technology (Beckman Coulter, Brea, CA) as described in 454 Technical Bulletin n. 2011–002 'Short Fragment Removal Procedure'. After clean-up, the products were quantified by both *Qubit* (LifeTechnologies, Carlsbad, CA) and qPCR using the KAPA Biosystems Library Quantification Kit (KapaBiosystems, Woburn, MA). Products were pooled based on molar amounts, run on a 1% agarose gel and extracted. After clean-up with a QIAquick PCR Purification kit (Qiagen, Valencia, CA) quality and quantity were assessed using a DNA 7500LabChip on the Agilent 2100 Bioanalyzer (Agilent Technologies, Santa Clara, CA) and *Qubit* quantification.

The sequencing was performed using a quarter PTP plate on a 454 Life Sciences Genome Sequencer FLX+ (Roche Diagnostics, Indianapolis, IN) per 454 specifications. One-way read amplicons (Lib-L) were prepared using the bar-coded fusion primers Forward: CCATCTCATCCCTGCGTGTCTCCGACTCAG-MID- Forward Specific Primer and Reverse: CCTATCCCCTGTGTGCCTTGGCAGTCTCAG-Reverse Specific Primer. PCR reactions (25 μl) for the 16s amplicons contained 5 pmoles of forward and reverse primers, dsDNA (10 to 40 ng), 5 nmoles of dNTP, 0.25 μl of TAQ (Fast Start High Fidelity PCR system, Roche, Indianapolis, IN), and 2.5 μl of 10X buffer supplied with the enzyme. Samples were denatured at 94°C for 3 min, then cycled (Gene AMP PCR System 9700; Life Technologies, Carlsbad, CA) 27 to 35 cycles at 94°C for 15 sec, 55°C for 45 sec and 72°C for 60 sec with a final extension at 72°C for 8 min. Samples with less than 20,000 reads were repeated aiming for an average of 40,000 read/sample. One Control+Collar rabbit did not provide enough DNA and was not included in the microbiota analysis. The genomic work was performed at the Genomics Core Facility of the Pennsylvania State University.

### Immune gene expression and antibodies

Tissue sections were also used to quantify the expression of cytokines/transcription factors/function genes using qRT-PCR and the somatic and the total mucus IgA using ELISAs [45]. Unless stated otherwise, all reagents and equipment were purchased from Thermo Fisher Scientific (Waltham, MA) and used as directed. In addition to the previously estimated genes for IFNγ, IL4 and IL10 [45] we also determined expression of the type 2 cytokines IL5 and IL13, the regulatory T cytokine TGFβ1, the transcriptional regulators Tbet, GATA3, Foxp3 and RORγT and the mucin MUC2 and MUC5AC. Primers and probes were synthesized using Taqman based qRT-PCR technology and target sequences using available genomic and transcriptomic data (Table A in S1 File) gathered from the Ensembl database (link: http://useast.ensembl.org/Oryctolagus_cuniculus/Info/Index). The linearity in amplification efficiency of all primer-probe sets was confirmed with 12 cDNA samples ranging from 0.1 to 100 ng, sourced from the duodenum sections (4 control and 8 infected) of previously collected samples [50]. Two technical replicates were performed for every sample.

The gene expression data were transformed using the comparative 2^-ΔΔCt^ method [51] and correcting the raw Ct values by the associated housekeeping gene HPRT and the related Ct averages from the four baseline rabbits sampled at day 0. These standardized data were then used for the statistical analyses.

ELISAs were performed to quantify *T*. *retortaeformis*-specific mucosal IgA as described in our previous work [45]. Total IgA concentration was quantified using a sandwich ELISA developed in-house (further details in Text A in S1 File). The amount of captured mucosal total IgA was extrapolated from known concentrations of purified IgA standards (Innova Biosciences, Cambridge, UK) also captured and detected with the same antibodies. The purified IgA standards were also used to confirm specificity of capture and detection antibodies for rabbit IgA. Four-fold dilutions of purified IgA standards were tested at an effective concentration range of 0.25–6.1x10^-5^ μg/ml. Mucus total IgA concentration was estimated at dilutions of 1:3000 and 1:9000.

### Parasitology

Helminths were collected from the duodenum samples of every animal and counted using aliquots following procedures described in our previous studies [45].

### Bioinformatics for microbiota composition and functionality

The 16S rRNA gene sequences were processed with MOTHUR metagenomics analysis tool [52] to produce a high quality data set. Sequences and their quality information were extracted from the binary files using the *sffinfo* command. First, we removed the primer and adapter contamination and second, the entire data set was subjected to quality trimming by checking for an average base quality of 35 within window size of 50 base, where a base quality of 30 means 99.9% base call accuracy. The resulting trimmed and quality filtered reads were aligned via Basic Local Alignment Search Tool (BLAST) [53] against the non-redundant 16S bacterial database collected at GenBank. The BLAST alignment E-Value 10 was chosen in order to generate many candidate alignments [53]. The taxonomic content of these resulting blast hits was then identified using the program MEGAN (METaGenome ANalyzer) [54] and the associated NCBI taxonomy. The classification by MEGAN allows us to identify the number of reads assigned to each taxonomic level: 89.5% of the data were classified into bacteria, or any of the bacterial taxonomic levels, while the rest fell into “No hits” or “Low Complexity” categories. These bacterial reads were then used in the subsequent statistical analysis.

We also characterized the functional properties of the microbiota using QIIME [55, 56] and PICRUSt [57]. The ‘pick_closed_reference-otus.py’ script in QIIME was used to search the sequences against GreenGenes (GG) reference OTUs at 97% identity. Reads that did not hit the reference collection were discarded. PICRUSt normalizes the generated OTU-table by dividing the read count for a given OTU in each sample by its predicted 16S rRNA copy number. The methods of PICRUSt then predict the functional content of the metagenome by multiplying the normalized OTU abundance in each sample by its pre-calculated gene-content abundance in KEGG gene families [58]. The predicted functional contents were collapsed at KEGG pathway hierarchy levels 1, 2 and 3 for interpretation and subsequent analyses.

### Statistical analysis

To assess the potential for sample size biases in the microbiota measurements, a rarefaction analysis was conducted initially where the fraction of taxa was captured for each rabbit using 100 bootstrapped samples of reads, taken at a range of sampling sizes from 1 to the maximum library size of every individual (Fig A in S1 File). Results show that we were able to consistently identify all the detectable taxa present in every animal with a minimum reading depth of 13,500 and 10,500 reads/sample at the phylum and family taxonomic level, respectively. We also performed a preliminary analysis to examine changes in microbial diversity and number of taxa by treatment and sampling time using both the raw and the rarefied data (all rabbits down-sampled to the library size N = 8,691, 7,178 or 4,666 for phylum, family and genus, respectively) and the two datasets showed statistically similar results. This suggests that the variation of the microbiota among animals is not likely caused by low sequence coverage. Therefore and in agreement with previous work [59], all the statistical analyses were performed using the raw data and the statistical software R (R core team, www.r-project.org).

To quantify the microbiota alpha diversity in the duodenum of every individual and to determine changes among treatments and sampling time, two diversity indices (Shannon Weaver and Simpson diversity) were examined at the phylum, family and genus level with the R package Vegan [60] and using a linear model ANOVA followed by the *post-hoc* Dunnett's Modified Tukey-Kramer pairwise multiple comparison test of mean differences. Linear model ANOVAs and *post-hoc* tests were also used to examine changes in the number of bacteria taxa among treatments and sampling time at the three taxonomic levels. To detect the most common taxa, the relative abundance of bacteria in every animal from the 4 treatments was visually examined at the phylum, family and genus level. To assess how the structural variability in the microbiota composition affected the clustering of animals by treatment, a principal coordinate analysis (PCoA, package Vegan) was performed using both the Bray-Curtis and the UniFrac distance matrix at the phylum, family and genus level. The variability of the principal coordinate components was quantified as the percentage of the eigenvalue corresponding to a specific component over the sum of eigenvalues from all the components considered. Treatments were visually clustered drawing ellipses estimated as the 95% confidence region of the joint distribution of the two components considered. PerMANOVAs and the Bray-Curtis distance matrix were used to examine changes in the abundance of microbial taxa by treatment and sampling time at the three taxonomic levels using the function Adonis in the package Vegan from the program R [60].

A linear regression model was used to identify differences in log-transformed immune response, or helminth abundance, among treatments and sampling times as well as between the immune response and parasite abundance. The linear regression residual plots were used to assess appropriateness of the model; outliers and influential observations (none of the two types were found) were screened using standard diagnostics [61]. PCoA and PerMANOVA were implemented to identify the clustering of individuals based on their immune profile and how they differed among treatments and sampling times. A Spearman rank correlation analysis was applied to identify trends in the relationship between microbiota abundance and immune response, or parasite abundance, within each treatment. Only the family taxa for which the coefficient of variation (CV) among rabbits was greater than 150 were selected for the analysis. Of the 124 families available, 22 were identified with CVs ranging between 12,269.78 and 167.34, compared to the remaining 102 families with CVs between 102.54 and 1. The correlation approach allowed us to avoid any causative assumption in the variation between bacteria abundance and immune variables (or parasite abundance), while emphasizing significant relationships and trends that could indicate either positive or negative interactions.

These analyses were repeated to examine the functional characteristics of the duodenum microbiota, how they vary over time and how these changes relate to the immune response, or parasite abundance. We used the metagenomic data re-classified based on the KEGG pathway functional module 2. PCoA and PerMANOVA were performed to highlight host variation in the functional activities by treatment and sampling time. The relationship between the host immune response, or the helminth abundance, and the KEGG level 2 was investigated using Spearman rank correlation analysis and selecting the functional activities for which the coefficient of variation (CV) among rabbits was greater than 70,000 (range 70,001–663,708), 20 out of 41 functions were selected.

## Results

### Characterization of the duodenal microbiota by treatment

**Fig 1:**
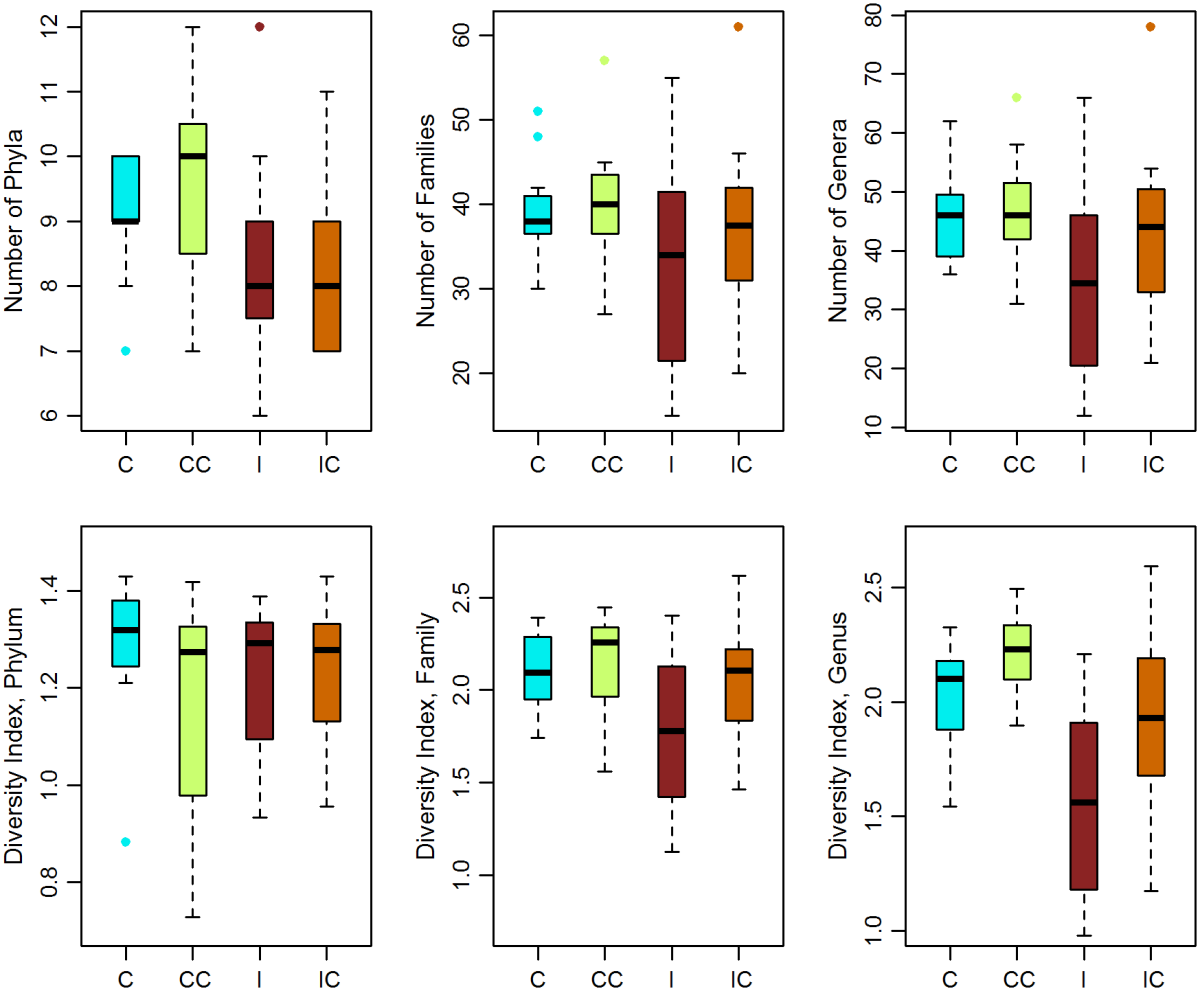
Abundance and alpha diversity of the duodenum microbiota. Abundance and diversity by treatment (Infect: I, Infect+Collar: IC, Control+Collar: CC and Control: C) at the 3 taxonomic levels (time points pooled together). The mean, 25–75% percentiles, maximum and minimum and outliers from the Shannon diversity index are reported. Diversity is significantly lower in I than in CC or C groups. The number of taxa is similar among treatments.

The alpha diversity and number of bacterial taxa were significantly different between treatments and sampling time at the family and genus but not the phylum level (Fig 1, Table B in S1 File). The *post-hoc* pairwise comparison test between treatments found a lower diversity in the Infect group compared to the Control (group difference: -0.437 C.I.: -0.867, -0.006) and the Control+Collar (group difference: -0.647, C.I.: -1.056,-0.236)at the genus level (for both, Dunnett's Modified Tukey-Kramer test: P<0.05); no significant differences were found for the number of bacterial taxa.

**Fig 2:**
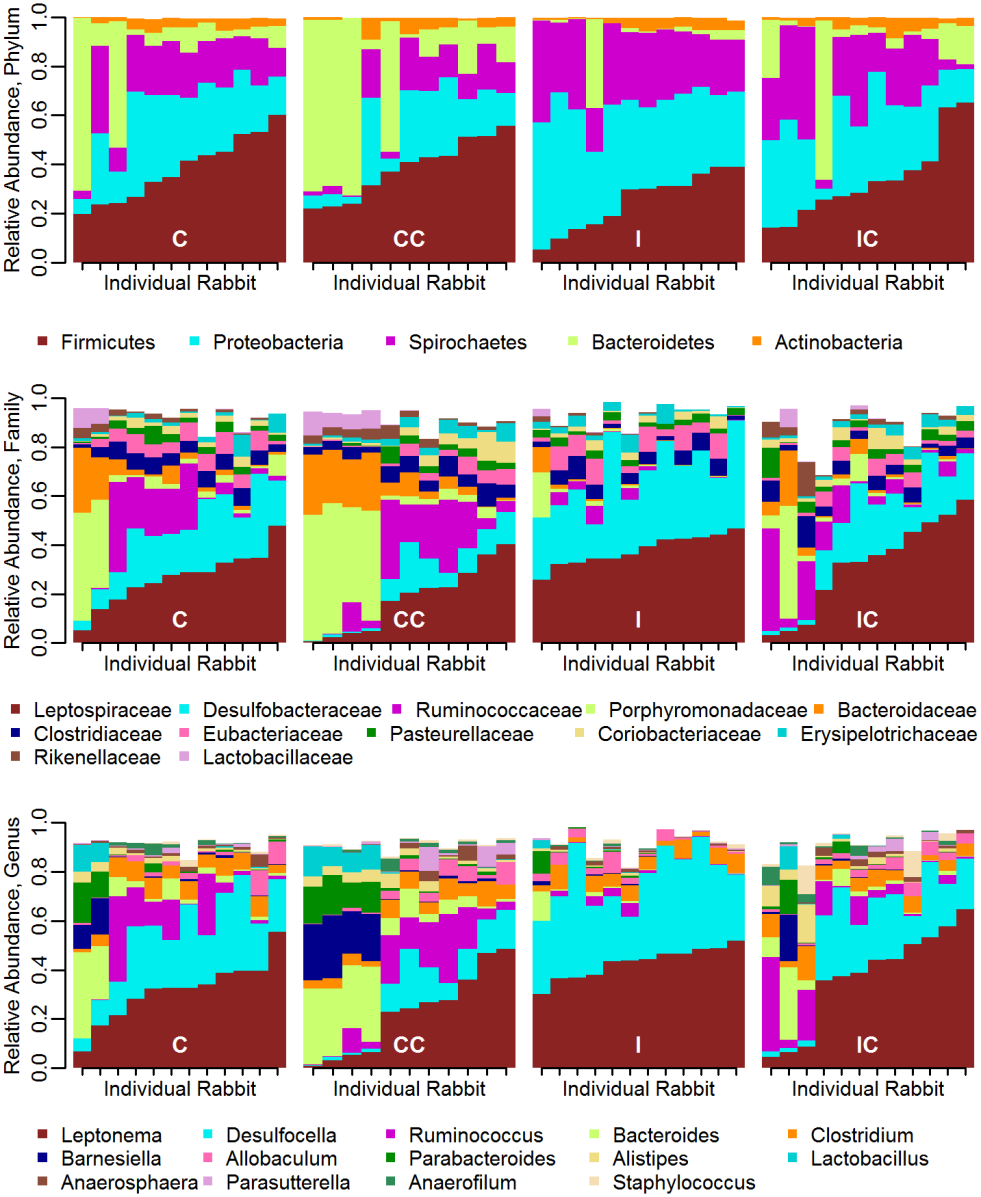
Relative microbiota abundance by animal. Abundance at the 3 taxonomic levels in the four treatments (Infect: I, Infect+Collar: IC, Control+Collar: CC and Control: C). Only the taxa with a relative abundance greater than 1% at each taxonomic level are presented. The white space that adds up to 100% abundance should be interpreted as 'other' taxa.

At the family level, *Leptospiraceae* (*Spirochaetes* phylum) and *Desulfobacteraceae* (*Proteobacteria* phylum) dominated in Infect and Infect+Collar cases whereas Control and Control+Collar groups also had the additional dominant families *Ruminococcaceae* (*Firmicutes* phylum), *Porphyromonadaceae* and *Bacteroidaceae* (both *Bacteroidetes* phylum) (Fig 2). This general trend was maintained at the genus level: *Leptomena* (*Leptospiraceae* family, *Spirochaetes* phylum) and *Desulfocella* (*Desulfobacteraceae* family, *Proteobacteria* phylum) were the most abundant in Infect and Infect+Collar individuals. Specifically, *Spirochaetes* were mostly characterized by *Leptonema illini*, a non-pathogenic *Leptospiraceae* also found in livestock and small-mammals [62] while *Proteobacteria* were mostly represented by *Desulfocella halophila*, a bacterium with fatty acid and butyrate oxidizing functions [63]. Control and Control+Collar cases tended to carry less of these genera but a higher percentage of the cellulolytic *Ruminococcus* (*Ruminococcaceae* family, *Firmicutes* phylum) and *Bacteroides* (*Bacteroidaceae* family, *Bacteroidetes* phylum) (Fig 2).

The principal coordinate analysis (PCoA) using both the Bray-Curtis and UniFrac distance matrix generated similar conclusions, therefore, only the results from the first approach are presented. The first two principal components explained more than 50% of the variation in the microbiota among animals at the phylum, family and genus taxonomic level (Fig B in S1 File). Using the Bray-Curtis distance matrix we found significant differences in the abundance of the different bacterial taxa by treatment at the three taxonomic levels (Table C in S1 File). The strongest differences were found between Control+Collar and Infect groups while Infect+Collar and Control individuals were more overlapped (Table D in S1 File).

In summary, the Infect group exhibited the less diversified and the Control+Collar the more diversified microbiota, Infect+Collar carried an 'in-between' community. Bacterial species contributing to fatty acid oxidizing functions appeared to be more common in infected cases.

### Characterization of the local host immune response by treatment

**Fig 3:**
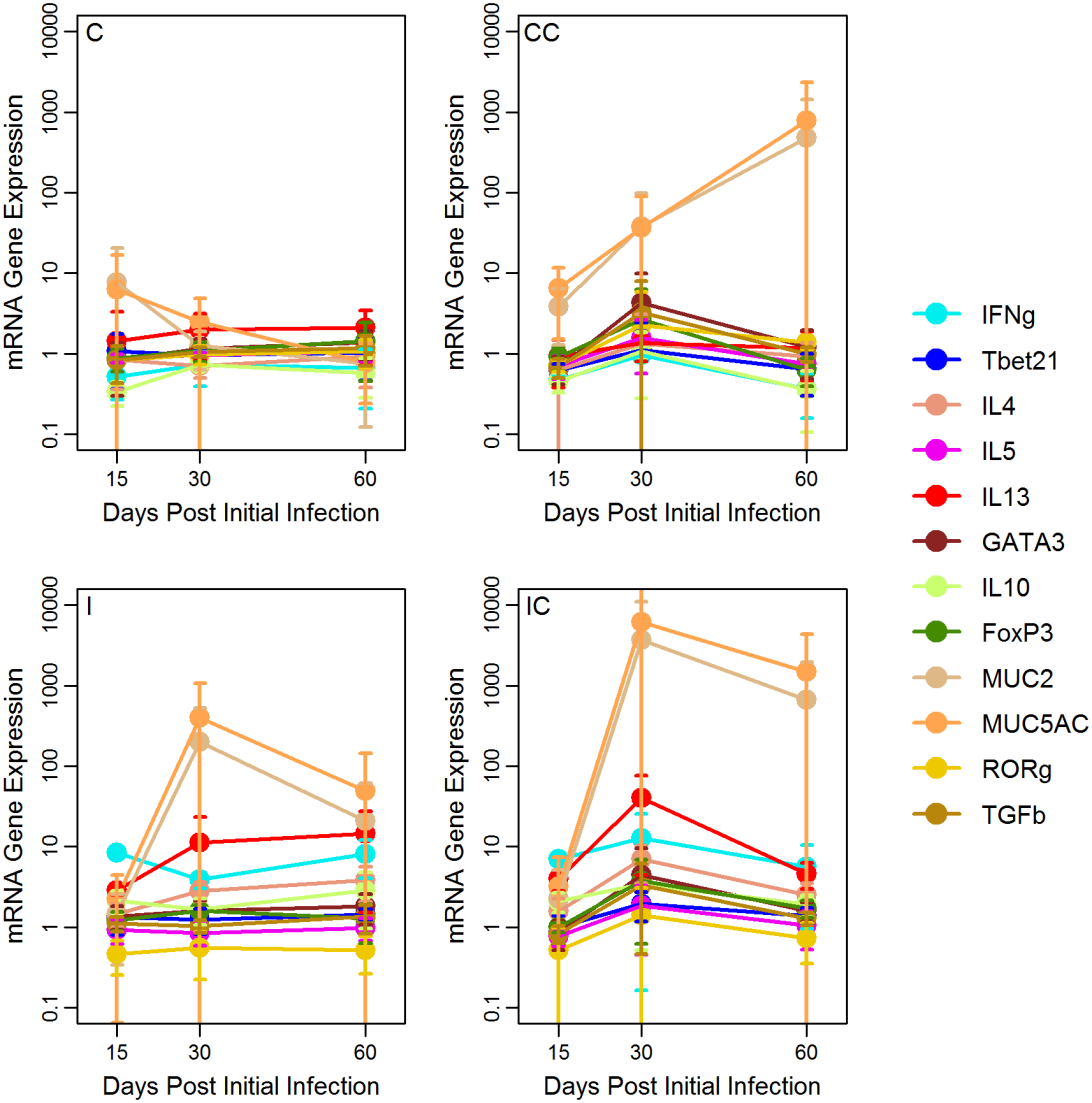
Expression of cytokine, transcription factor and function genes in the duodenal mucosa. Mean values (2^-ΔΔct^±s.e.) by treatment (Infect: I, Infect+Collar: IC, Control+Collar: CC and Control: C) and sampling time (day post initial infection). Data have been standardized by the housekeeping gene HPRT and the mean values from the baseline animals sampled at day 0. Most of the variables show significant differences among treatments but not sampling time, higher values are observed in the I and IC groups.

**Fig 4:**
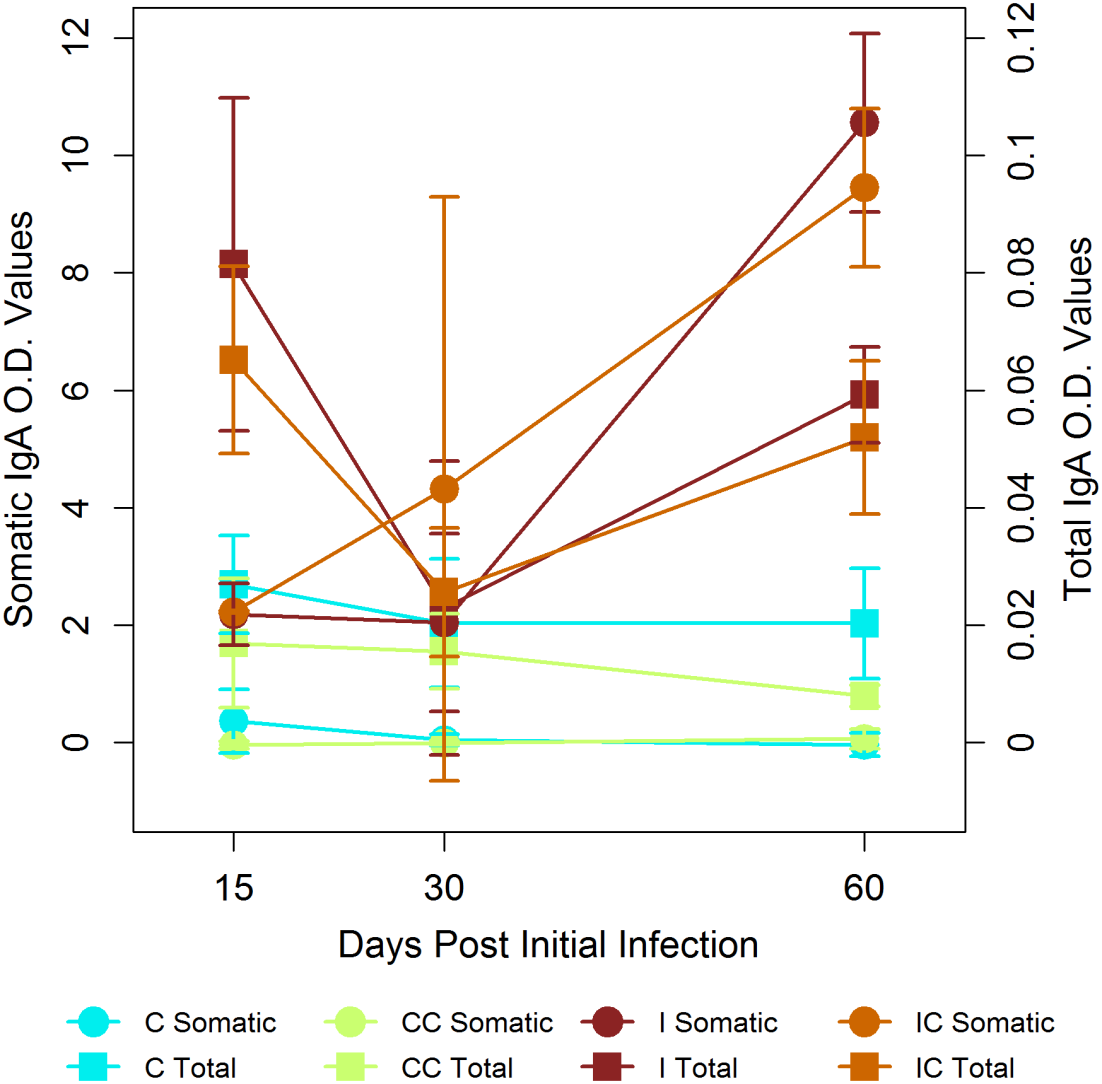
Antibody IgA from the duodenal mucus. Mean (±s.e.) Optical Density (O.D.) for the species-specific (left y-axis, full cycle) and the total (right y-axis, full square) IgA by treatment (Infect: I, Infect+Collar: IC, Control+Collar: CC and Control: C) and sampling time (days post initial infection). Both specific and total IgA are higher in I and IC than in CC or C.

The majority of cytokines/transcription factors were significantly different among treatments but not sampling time (Fig 3, Table E in S1 File). *Post-hoc* pairwise comparison tests between treatments showed that gene expressions were significantly higher in Infect and Infect+Collar groups compared to Control and Control-Collar cases (Table F in S1 File). A type 1-type 2 immune response was observed in the duodenum of Infect and Infect+Collar animals: IFNγ and IL13 dominated the immune profile followed by a much lower expression of IL4, IL10, GATA3 and FoxP3. Control+Collar individuals exhibited a low to very low levels of expression of the genes examined. Mucus gene expression, MUC5 and MUC2, was highly variable over time except in the Control cases, which showed baseline values; yet, no differences were observed among treatments (Fig 3). Both *T*. *retortaeformis*-specific and total IgA from the duodenal mucus were significantly different among treatments and over time (Fig 4, Table E in S1 File). As expected from our previous work [45, 50], specific IgA was higher and increased with time in the infected groups; total mucus IgA was also high in infected cases while the non-infected groups exhibited the lowest values (Table F in S1 File).

The PCoA analysis of the individual immune profile by treatment explained 57% of the variation observed with the first two principal components (Fig C in S1 File). Significant differences were found among treatments (PerMANOVA using Bray-Curtis matrix: gene expression by treatment, SS, df, p: 1.94, 3, <0.001; antibody level by treatment p<0.001, sampling time and their two-way interaction, for both p<0.01), infected groups tended to cluster more closely together as did the control rabbits (Table G in S1 File).

Overall, infected animals up-regulate immune genes contributing to the control of the helminth infection as well as genes involved in inflammatory responses. The immune profile of Control+Collar cases is more similar to the baseline Control group.

### Relationship between microbiota and host immunity

**Fig 5:**
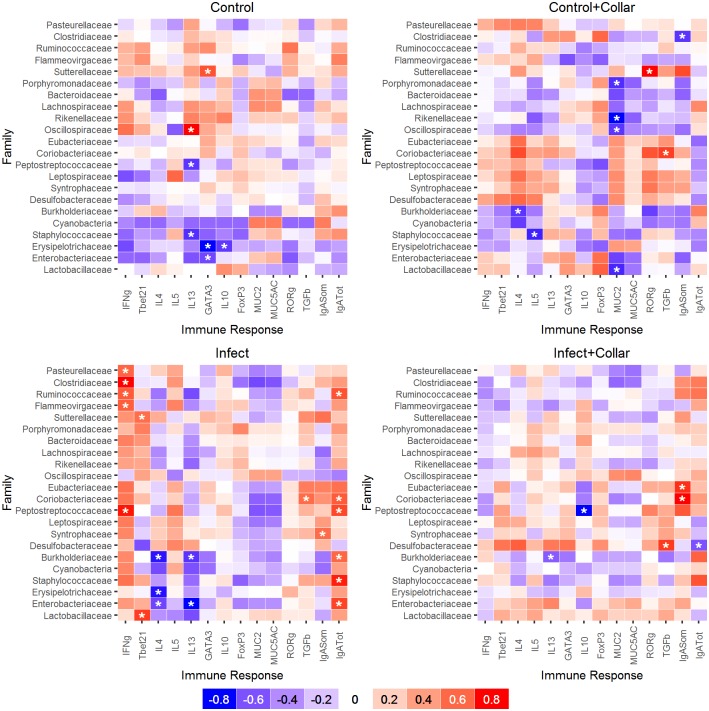
Relationships between microbiota and immune response. Spearman rank correlations grouping animals by treatment (Infect: I, Infect+Collar: IC, Control+Collar: CC, and Control: C) and using only the bacteria that showed a between-individual coefficient of variation (CV) greater than 150. Negative relationships are in grades of blue while positive relationships are in grades of red; a correlation coefficient quantifies each grade, null correlations (0) are depicted in white; significant correlations (p<0.05) are highlighted with a star. Significant correlations are more common in the Infect group than the remaining treatments.

Our goal here was to identify whether changes in bacterial abundance were associated with the immune response and whether these changes were related to a specific treatment. A number of significant correlations were identified between the bacteria families whose abundance exhibited a coefficient of variation among the rabbits greater than 150 (CV>150) and variation in the immune response (Fig 5). In the Infect group, positive relationships were found between IFNγ gene expression and the abundance of *Pasteurellaceae*, *Clostridiaceae*, *Ruminococcaceae*, *Peptostreptococcaceae* and *Flammenovirgaceae*; positive relationships were also found between Tbet and *Lactobacillaceae* or *Sutterellaceae*, and between TGFβ and *Coriobacteriaceae*. Total IgA was positively related to *Enterobacteriaceae*, *Staphylococcaceae*, *Coriobacteriaceae*, and *Ruminococcaceae* among some. In contrast, expression of IL13 and IL4 was negatively related to *Enterobacteriaceae* and *Burkholderiaceae*. Few significant associations were found in the Infect+Collar individuals specifically: *Desulfobacteriaceae* were related positively to TGFβ and negatively to total IgA, negative associations were also found between IL13 and *Burkholderiaceae* and IL10 and *Peptostreptococcaceae*. In the Control+Collar group, negative associations were found between IL4 and *Burkholderiaceae*, IL5 and *Staphylococcaceae* and between MUC2 and few families including *Lactobacillaceae*. Few negative associations with IL13, GATA3 or IL10 were observed in the Control group.

All together, in the infected animals some bacteria families decrease while others increase with the course of the infection but whether this is affected by, or contributes to, the activity of specific immune variables is difficult to disentangle. For the collared animals, few bacteria families appear to be associated with a type 2 immune reaction or mucus production.

### Relationships between microbiota and intensity of infection

**Fig 6:**
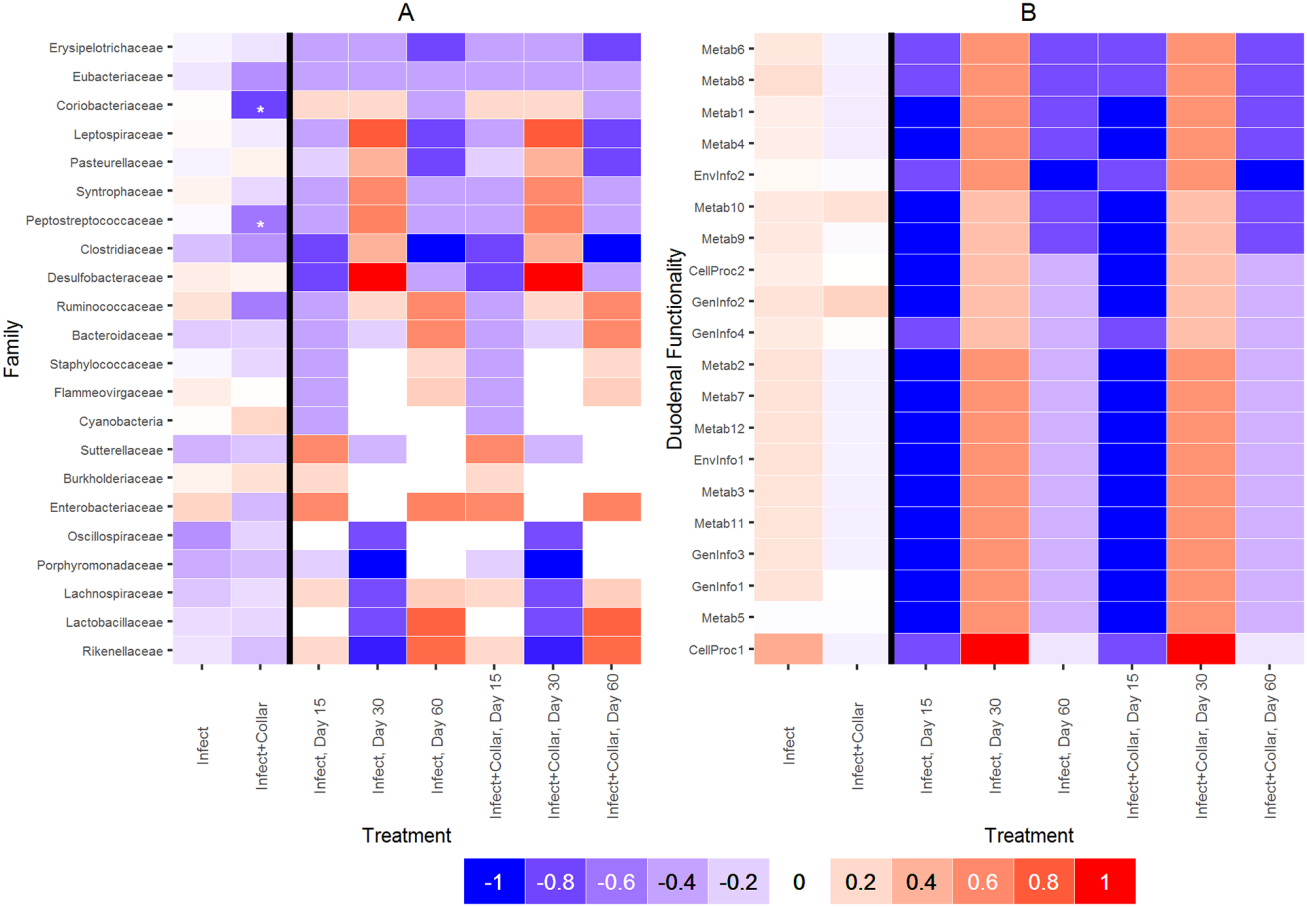
Relationships between microbiota and *T*. *retortaeformis* abundance. Spearman rank correlations grouping animals by treatment (Infect -I, Infect+Collar -IC), or treatment and time point, and: Panel A- the bacteria families with CV>150 or Panel B- the functions at the KEGG pathway module 2 that exhibited a coefficient of variation CV among individuals greater than 70,000. Additional details on the color-coding of the correlations are reported in Fig 5, while the activities related to the KEGG function IDs are listed in Fig 7. No significant correlations are detected, except in two cases.

The impact of the *T*. *retortaeformis* infection on the duodenal microbiota was examined in the Infect and Infect+Collar treatments. No significant relationships were found between parasite abundance and bacterial alpha diversity, or abundance, once the effect of treatment and time were taken into account. A correlation analysis between parasite abundance and the bacteria families with a coefficient of variation (CV) among rabbits greater than 150 showed negative associations of *Coriobacteriaceae* and *Peptostreptococcaceae* with parasite abundance in the Infect+Collar treatment (Fig 6A). Once sampling time was explicitly considered we found clusters of families with either positive or negative, albeit not significant, correlations at 30 days post initial infection in both treatments, which coincides with a reduction of the average parasite burden in the Infect group but no changes from the 15 day sampling point in Infect+Collar (Fig 6A and Fig D in S1 File).

In summary, while there are notable trends between a number of bacteria families and the intensity of infection, these relationships are not statistically significant.

### Relationships between helminth infection and host immunity

Given that *T*. *retortaeformis* is controlled by host immunity [45, 50] we examined the relationship between intensity of infection and the local immune response, and whether a restriction from cecotrophy affected the parasite load. We found no significant differences in the intensity of infection between treatments (Infect -I and Infect+Collar -IC) and sampling time (Fig D in S1 File); this trend was also confirmed in the *post-hoc* comparison test. Based on this finding, a linear model combining the two treatments together showed that parasite abundance was related positively with IFNγ (coeff.: 0.483) and GATA3 (3.170) (for both: p<0.01) and negatively with Tbet (-6.142, p<0.01), IL5 (-3.497) and MUC2 (-0.001) (for both: p<0.05).

Overall, the mean intensity of infection is similar between Infect and Infect+Collar and in both groups parasite abundance is associated to a mixed type 1-type 2 immune response.

### Characterization of the duodenal functionality by treatment

**Fig 7:**
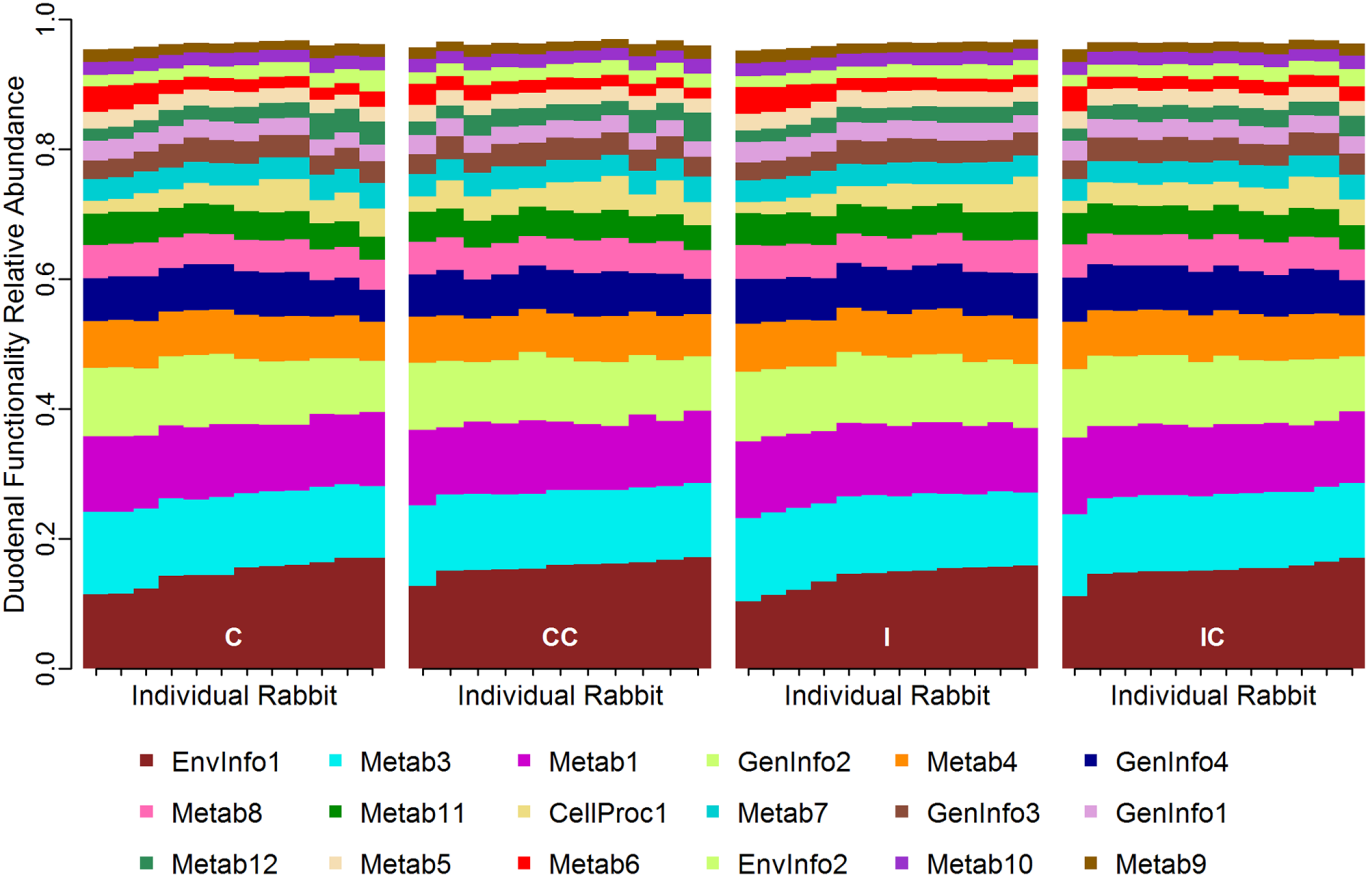
Relative abundance of gene functionality in the duodenum by animal. Data are presented by treatment (Infect: I, Infect+Collar: IC, Control+Collar: CC, and Control: C) at the KEGG pathway level 2. Only the functions with a relative abundance greater than 1% were displayed. The white space that adds up to 100% abundance should be considered as 'other' functions. The following function IDs and related activities were selected: CellProc1 = Cellular Processes: Cell Motility; CellProc2 = Cellular Processes: Transport and Catabolism; EnvInfo1 = Environmental Information: Processing Membrane Transport; EnvInfo2 = Environmental Information: Processing Signal Transduction; GenInfo1 = Genetic Information: Processing Folding Sorting and Degradation; GenInfo2 = Genetic Information: Processing Replication and Repair; GenInfo3 = Genetic Information: Processing Transcription; GenInfo4 = Genetic Information: Processing Translation; Metab1 = Metabolism: Amino Acid Metabolism; Metab2 = Metabolism: Biosynthesis of Other Secondary Metabolites; Metab3 = Metabolism: Carbohydrate Metabolism; Metab4 = Metabolism: Energy Metabolism; Metab5 = Metabolism: Enzyme Families; Metab6 = Metabolism: Glycan Biosynthesis and Metabolism; Metab7 = Metabolism: Lipid Metabolism; Metab8 = Metabolism: Metabolism of Cofactors and Vitamins; Metab9 = Metabolism: Metabolism of Other Amino Acids; Metab10 = Metabolism: Metabolism of Terpenoids and Polyketides; Metab11 = Metabolism: Nucleotide Metabolism; Metab12 = Metabolism: Xenobiotics Biodegradation and Metabolism.

**Fig 8:**
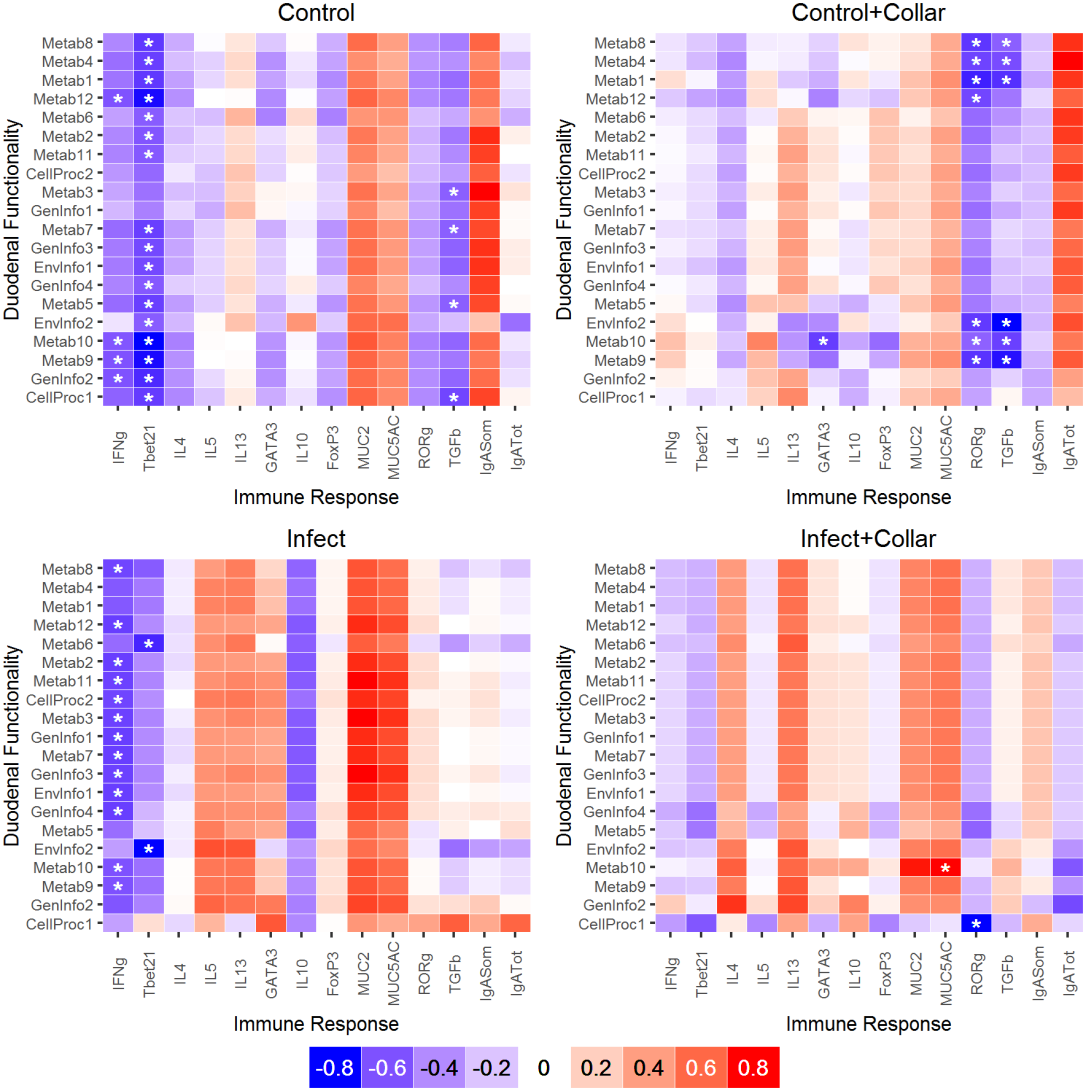
Relationships between gene functionality and immune response. Spearman rank correlations grouping animals by treatment (Infect: I, Infect+Collar: IC, Control+Collar: CC, and Control: C) and using the KEGG pathway level 2 functions with a CV>70,000. Additional details are reported in Figs 5 and 7. Significant correlations are mainly observed with a type 1 response.

To examine if the helminth infection and the cecotrophic restriction affected the functional and metabolic properties of the duodenal microbiota, and how these changes related to the immune response, analyses were repeated using the KEGG pathway functional module 2. The functionality of the bacterial community was similar between treatments (*post-hoc* analysis) and consistent among animals (PCoA and PerMANOVA approach) (Fig 7 and Fig E in S1 File, Table H in S1 File). The most common functions were pathways of replication/repair and membrane transport, and metabolic activities such as carbohydrate, amino acid and energy metabolism. This general pattern was also consistent at the KEGG pathway functional module 3 (data not shown). The correlation analysis between the most variable pathways among individuals (CV> 70,000) and their immune response identified general trends that were consistent among treatments, with few significant correlations (Fig 8). Specifically, metabolic pathways were negatively correlated with IFNγ and Tbet gene expression in Infect and Control treatments and with RORγ and TGFβ in Control+Collar and Control.

Positive, although non-significant associations between intensity of infection and microbiota functionality were consistently detected at 30 days post initial infection (Fig 6B), as reported for the bacterial abundance.

These findings suggest that while some clear trends were detected between the majority of the immune variables and the microbiota functionality in every treatment, few were statistically significant.

## Discussion

We initially proposed that *T*. *retortaeformis* infections altered the composition and abundance of the microbiota in the duodenum, and associated changes in the immune response. These modifications were expected to become more obvious by preventing the rabbits from absorbing fundamental nutrients via cecotrophy.

Findings suggest that both the helminth infection and the nutritional restriction affected the microbial community, although the impact led to contrasting outcomes among the treatments. In the Infect group we found a reduction both in the overall diversity and abundance of the microbiota, and a few species appeared to prevail some phyla. For example, *Spirochaetes* were dominated by the *Leptonema illini*, also found in cattle, pigs and small-mammals [62] while *Proteobacteria* were mostly represented by *Desulfocella halophila*, a bacterium with fatty acid and butyrate oxidizing functions [63]. The fatty acid metabolism is particularly relevant because it produces large yields of ATP and is important for membrane formation and signaling pathways [64]. Rabbits need more energy to fight the infection but also to repair the mucosa damaged by the parasites moving across the tissue and *D*. *halophila* might play an important role in these activities.

Surprisingly, Control+Collar cases exhibited the most diversified and abundant microbiota. Animals carried less *D*. *halophila* but higher proportion of *Firmicutes*, such as the highly cellulolytic *Ruminococcaceae* and the families *Porphyromonadaceae* and *Bacteroidaceae* both involved in glucose and fat metabolism. *Bacteroidetes* are often associated with obese phenotypes and a microbiome from diets high in proteins and fat [65–67]. The proliferation of *Bacteroidetes* in the duodenum could indeed be related to the type of pellet used for the rabbit, which consisted of 21% proteins and fat (see Materials and Approach). We propose that by reducing the availability of ready-to-absorb compounds, the cecotrophic prevention appears to have stimulated a more diversified microbiota, particularly the proliferation of bacteria that can facilitate nutrients intake from less digested food. This can also explain previous work that found an increased digestive activity in rabbits prevented from cecotrophy [68]. Rabbits' soft feces (cecotropes) have a distinct microbial structure but a similar richness compared to hard feces [69], suggesting that the ingestion of cecotropes can help to re-establish and diversify, but not to enrich, a depleted gut microbiota. Our experiment shows that both diversity and abundance do increase in the small intestine once the ingestion of soft feces is prevented.

In general, Infect+Collar animals harbored a microbiota "in-between" the Infect and the Control+Collar. These findings support the hypothesis that hosts need a bacterial community that can generate large yields of high quality energy (i.e. fatty acid oxidizing bacteria) to cope with the helminth infections. However and contrary to our prediction, the collar did not exacerbate the severity of the infection, instead, Infect+Collar animals appear to control the parasites as well as the infected cases.

This work is consistent with previous laboratory studies that found an impoverishment of the gut microbiota with helminth infections [7, 8, 10] but contrasts with recent field studies that showed an increase in bacterial diversity among parasite positive individuals from rural settings in developing countries [11] or a natural population of mice co-infected with different helminth species [70]. Multiple factors could have contributed to these contrasting outcomes. A pattern that seems to emerge is that a simplified diet is often associated with a highly diversified microbiota that facilitates the nutritional intake; a trend frequently observed in rural areas from developing countries or hunter/gatherer societies [14, 15]. A diversified microbiota from a simplified diet has been suggested to protect from gut diseases [15] and it can also contribute to mitigating the severity of gastro-intestinal helminth infections. The general agreement from studies on helminth-microbiota interaction is that, whether the parasite leads to a depletion/amelioration or a shift of the bacterial community, changes appear to be associated with the characteristics of the parasite (i.e. species, intensity of infection and duration of the exposure) and the host (i.e. the infected organ and the tissue/material selected for the analyses). Our experiment shows that trickle doses of *T*. *retortaeformis*, comparable to natural infections [43], reduced the microbiota diversity although few species with critical properties appeared to dominate. In contrast, nutritional restrictions increased the bacterial diversity despite the parasite infection.

Changes in the bacterial abundance (based on the families with the greatest variability between animals) were associated with changes in the local immune response. However, whether the immune response was primarily driven by the direct reaction of the host or partly mediated by the microbiota could not be disentangled with the current experiment. The immune profile of Infect cases was consistent with our previous work by showing a mixed type 1- type 2 defensive response [45]. Three general patterns were observed with the microbiota. First, we found a number of positive associations with IFNγ, probably as a response to the bacteremia of the mucosa and/or the development of pathobionts following the damage of the duodenal wall by the parasites. Second, we recorded a number of negative relationships with the type 2 response, mostly IL13, IL 4 and GATA 3. While this immune reaction was targeting the helminth it is possible that it was also controlling the bacteria as a consequence of the worm infection. Third, we observed a weak association with a tolerogenic activity, whether it was positive (TGFβ, IL10) or negative (FoxP3), which is consistent with the evidence that the *T*. *retortaeformis*-rabbit interaction is predominantly a resistant phenotype [43, 50].

These general trends were weakly recapitulated in the Infect+Collar group and contrast with our original prediction that a combination of parasites and nutritional restrictions would have exacerbated the immune reactions and the relationships with the bacterial community. Indeed, we found no clear trends with the type 2 response and weak negative relationships with the type 1 (IFNγ, Tbet) and the regulatory (IL10, FoxP3) reaction. The lack of a clear immune-microbiota trend could be indicative of better local conditions compared to Infect cases, for example, animals could be more successful in coping with an inflammatory response. For the Control+Collar cases we also found no clear relationships with the immune response.

The functional activities of the duodenum microbiota remained relatively conserved among the treatments, and mostly targeting metabolic and membrane transport activities. Significant negative relationships were found between the microbiota functionality and the expression of IFNγ and Tbet in Infect and Control treatments, implying that some critical functions are deregulated or suppressed under inflammatory conditions. For example, previous work showed that the loss of Tbet was associated with spontaneous inflammation that could be cured with antibiotics, suggesting the involvement of the microbiota [71]. This could explain the negative relationships with Tbet and the need to maintain a balanced microbiota in Control cases. Similarly, IFNγ-T cells are produced from microbiota signals in steady state condition [72]; in our Control group this can be seen as a way to self-control by the bacterial community from excessive activities. In contrast, the high expression of IFNγ against the helminths in the Infect group appears to be mostly host-driven and indirectly affecting the bacterial functions. The lack of these trends in collared animals further supports the possible anti-inflammatory role of a more diversified microbiota from a simplified diet, in our case biased towards the absorption of nutrients from a fiber-rich diet with no cecotrophic reabsorption. Functions related to mucus production showed a tendency to increase with a type 2 response, which supports previous studies on the positive role of the microbiota in the type 2 immune defenses [6] and possibly in mucus production [73].

Our factorial experiment identified differences in the microbiota and immune response among treatments but not, or weakly, over time. Specifically, the bacterial community did not significantly change with the trickle dosing of *T*. *retortaeformis* or wearing the collars for a prolonged period of time. The lack of a significant relationship with parasite abundance was probably caused by using doses that resembled natural infections and prevented extreme reactions in the duodenum. Similarly, wearing the collars up to 60 days did not appear to have deteriorated the local gut conditions, indeed, all the animals consistently gained body mass over the course of the experiment irrespective of the treatment (Fig F in S1 File). However, we do not exclude that the small number of replicates and variation among groups affected some of the results. Sample size could also have influenced the lack of significant patterns in the functional metagenome analysis, although it could also be associated with the limitations of PICRUSt to process non-human data [69].

In conclusion, this study suggests that while the duodenal microbiota was depauperated by the *T*. *retortaeformis* infection, the local conditions did not exacerbate with the progression of the infection. At the end of the trial, the weekly dosing of parasites was partially controlled by the immune response, consistent with our previous work [43, 45, 74], and the bacterial community did not significantly change over time. Animals with both infection and cecotrophic restriction exhibited a more diversified and abundant microbiota, and a parasite load similar to the Infect cases. The collar enhanced bacterial diversity and abundance suggesting that the microbiota has to adjust to increase the absorption of nutrients from a simplified food. These findings provide important information to the understanding of how the gut microbiota adapts to the combined effect of helminth infections and nutritional alterations and how these components can be related to the local immune profile. Yet, more work is needed to clarify these relationship and the mechanisms driving these patterns. The *T*. *retortaeformis*-herbivore model has many similarities with parasite systems of livestock and humans and this study offers some general understanding that can be used to generate new hypotheses on the parasite-nutrition-microbiota interactions and ultimately improve individual health. Indeed, a tantalizing idea is that by choosing a correct diet it is possible to promote not only a more diversified bacterial community but also the proliferation of critical bacteria important for mitigating the impact of helminth infections. The next challenge is to identify the bacteria involved in the processes of parasite control, either directly or by influencing the immune response, and pinpoint ways to manipulate bacterial abundance by selecting a targeted diet that can promote bacterial diversity and/or proliferation of critical species important for alleviating infection severity.

## Supporting Information

S1 File **Text A**. Quantification of total IgA antibodies. **Table A**. Primer and probe sequences for the immune variables. **Table B**. Bacterial diversity index and abundance by treatment. **Table C**. Variability in the abundance of the bacterial community by treatment and sampling time. **Table D**. Elliptical distances of the PCoA in Figure B in S1 File. **Table E**. Variability of every immune variable by treatment and sampling time. **Table F**. *Post-hoc* pairwise comparison of the immune variables between treatments. **Table G**. Elliptical distances of the PCoA in Figure C in S1 File. **Table H**. Elliptical distances of the PCoA in Figure E in S1 File. **Figure A**. Rarefaction Analysis. **Figure B**. PCoA plots of the microbiota by treatment. **Figure C**. PCoA of the rabbit immune response by treatment. **Figure D**. *T*. *retortaeformis* abundance by treatment and time post initial infection. **Figure E**. PCoA of the duodenal microbiota functionality by treatment. **Figure F**. Rabbit body mass by treatment and time post initial infection.
(PDF)
