# Supplementary material for: Impact of Helminth Infections and Nutritional Constraints on the Small Intestine Microbiota
Source: PLoS One. 2016 Jul 20;11(7):e0159770. doi: 10.1371/journal.pone.0159770 (PMC4954658; doi:10.1371/journal.pone.0159770)

SUPPLEMENTARY MATERIAL

IMPACT OF HELMINTH INFECTIONS AND NUTRITIONAL CONSTRAINTS ON THE SMALL INTESTINE MICROBIOTA

Cattadori IM, Sebastian A, Hao H, Katani R, Albert I, Eilertson KE, Kapur V, Pathak A, Mitchell S

The following supplementary material is included:

Text A. Quantification of total IgA antibodies
Table A. Primer and probe sequences for the immune variables
Table B. Bacterial diversity index and abundance by treatment
Table C. Variability in the abundance of the bacterial community by treatment and sampling time
Table D. Elliptical distances of the PCoA in Figure B
Table E. Variability of every immune variable by treatment and sampling time
Table F. *Post-hoc* pairwise comparison of the immune variables between treatments
Table G. Elliptical distances of the PCoA in Figure C
Table H. Elliptical distances of the PCoA in Figure E
Figure A. Rarefaction Analysis
Figure B. PCoA plots of the microbiota by treatment
Figure C. PCoA of the rabbit immune response by treatment
Figure D. *T. retortaeformis* abundance by treatment and time post initial infection
Figure E. PCoA of the duodenal microbiota functionality by treatment
Figure F. Rabbit body mass by treatment and time post initial infection

Table A. Primer and probe sequences in 5’-3’ format used for gene expression assays from rabbit (*Orytolagus cuniculus)*. The gene expression assay for *TGF-β1* (ID provided), was available made-to-order from the manufacturer (Thermo Fisher Scientific, Waltham, MA). All probes were synthesized such that the 5’ end was conjugated to a reporter dye (Fluorescein amidite or FAM) and the 3’ end to a non-fluorescent quencher (NFQ) followed by the minor groove binding moiety.

Gene	*Forward primer*	*Probe*	*Reverse primer*
Foxp3	GCCATCCGCCACAACCT	CCTGCACAAGTGCTTC	CCCTTCTCACTCTCCACTCTCA
GATA3	CCCGGCGGTCCTCAAC	CCGGACACGCACCACC	GCCGGGTCCATGTACGA
IL-13	TGACAGGCAGCGTGTACTG	CAGCCCTGGAGTCCCT	GTTGCAGCCAGAGACATTGAC
IL-5	TCTTGGAGCTGCCTATGTTTGTG	CTCATGCGGATTTCTG	CAGGGTCTCTTTCACCACTGT
MUC2	GGGTGATGACCAACCAGGT	CTTGCCGTTGAAAATG	CGATGCCGTCCTTCTCGAA
MUC5AC	GCGCCTGCACCTACAAC	CAGGAGCCAAATACTC	GCACTCGGTGCAGTCTGT
RORγt	GCAGCGTTCCAACGTCTTC	CCACACGGACTGCCTC	CGCAGCGTTCCCACATCT
T-bet	GCCGGAGGCAGCGT	ACACGCACGTCTTCAC	CGATGAACTGCGTCTCTTGGAA
TGF-β1	Thermo Fisher Scientific assay ID = Oc04176122_u1		

Text A- Quantification of total IgA antibodies

A 'sandwich' ELISA was developed in-house to quantify total IgA concentrations in mucus collected from the small intestine duodenum, the primary site of colonization for *Trichostrongylus retortaeformis*. Optimizations and experimental details described herein were designed following procedures available from Thermo Fisher Scientific (Waltham, MA) in "Tech tip #65: ELISA technical guide and protocols", link: https://tools.lifetechnologies.com/content/sfs/brochures/TR0065-ELISA-guide.pdf). Rabbit IgA was captured from the mucus extracts by goat antibodies that selectively bind the heavy (alpha) chain of rabbit IgA (Abcam plc, Cambridge, MA, USA), prior to detection with mouse antibodies that bind the light chains of rabbit immunoglobulins (Jackson Immuno-research, West Grove, PA, USA). Since both antibodies bind physically distinct sites of rabbit IgA, any possibility of interference from shared binding sites was minimized. The amount of captured mucosal IgA was extrapolated from knowing the concentrations of purified IgA standards (Innova Biosciences, Cambridge, UK) captured and detected with the same antibodies. The purified IgA standards were also used to confirm specificity of capture and detection antibodies for rabbit IgA. Capture antibodies bound purified IgA but not IgG (BD Biosciences, Franklin Lakes, NJ) while detection antibodies do not bind in the absence of rabbit IgA (data not shown). Ideal antibody and reagent concentrations were determined by the checkerboard titration method as described comprehensively elsewhere (https://tools.lifetechnologies.com/content/sfs/brochures/TR0065-ELISA-guide.pdf). Capture antibody (15 µl of 0.094 µg/ml goat anti-rabbit IgA, v/v) diluted in 0.2 M carbonate/bicarbonate buffer pH 9.6 (v/v) was used to coat 384-well ELISA plates (Microlon® 600, Greiner Bio-One, Monroe, NC, USA) overnight at 4°C. When stored at 4°C in humidified chambers, plates with immobilized capture antibody showed no detectable loss in binding capacity for rabbit IgA for at least a week (data not shown). All steps were performed at room temperature unless stated otherwise. Plates were washed once and incubated with blocking buffer consisting of 2% Bovine Serum Albumin (BSA, Fraction V, EMD Millipore, Billerica, MA) dissolved in PBS-T (w/v) for 2 hours. Plates were washed once with PBS-T before adding IgA standards (v/v) and mucus samples (v/v) diluted in the same blocking buffer. Four-fold dilutions of purified IgA standards were added at an effective concentration range of $0.25\text{-}6.1\times10^{-5}$ µg/ml. Mucus samples with unknown IgA concentrations were added at the indicated dilutions (1:3000 and 1:9000). After overnight incubation at 4°C, plates were washed 4 times with PBS-T before detection of captured rabbit IgA with biotin-conjugated mouse anti-rabbit light chain antibodies, diluted 1:10,000 (v/v) in 0.4% BSA prepared in PBS-T (B+PBS-T, w/v), for 1 hour at room temperature. Plates were washed 3 times with PBS-T before the addition of the signal detection enhancer, polymeric HRP-conjugated streptavidin (Thermo Fisher Scientific, Waltham, MA), also diluted in B+PBS-T (1:10,000, v/v). An hour later, plates were washed 3 times with PBS-T and once with PBS before being developed with TMB colorimetric peroxidase substrate (KPL Inc., Gaithersburg, MD, USA) for 10 minutes as described in the previous section. The assay consistently resolved IgA concentrations ranging from 1 to 6.1×10^{-5} µg/ml. Optimum dilutions for the rabbit mucus were established by testing mucosal extracts collected at various stages post-infection from this and previously performed studies (Murphy et al. 2011, ref. 45 in the main text).

Figure A. Rarefaction Analysis. Each line represents the rarefaction curve for a particular rabbit (multiple lines/rabbits might overlap with each other) belonging to a particular treatment (Infect: I, Infect+Collar: IC, Control+Collar: CC, and Control: C). These curves illustrate the average percentage of taxa discovered in a given rabbit (y-axis) as a function of the sample size (x-axis). The vertical dashed lines are placed at the smallest library size (phylum N= 8,691, family N= 7,178) of the 16 rabbits. The curves are generated by subsampling *n* samples from a rabbit's number of reads, N, where N is the library size. Each curve ends at the rabbit library size. We took a sample of size *n* 100 times to observe the average, or expected, percentage of taxa present at that sample size for that rabbit.

From the curves below we see that the percentage of taxa detected levels off for all rabbits around the smallest library size indicated by the vertical line. This suggests that additional or deeper sampling does not add significantly to the diversity or percentage of taxa detected in rabbits with larger library sizes. It also indicates that differences in library sizes are not a significant contributor to observed differences in diversity level for our study. Indeed, we conducted preliminary analysis to examine microbial diversity and number of taxa by treatment and sampling time using the rarefied samples and the conclusions were consistent with the analysis based on the full library data (also see main text section: Statistical Analysis).

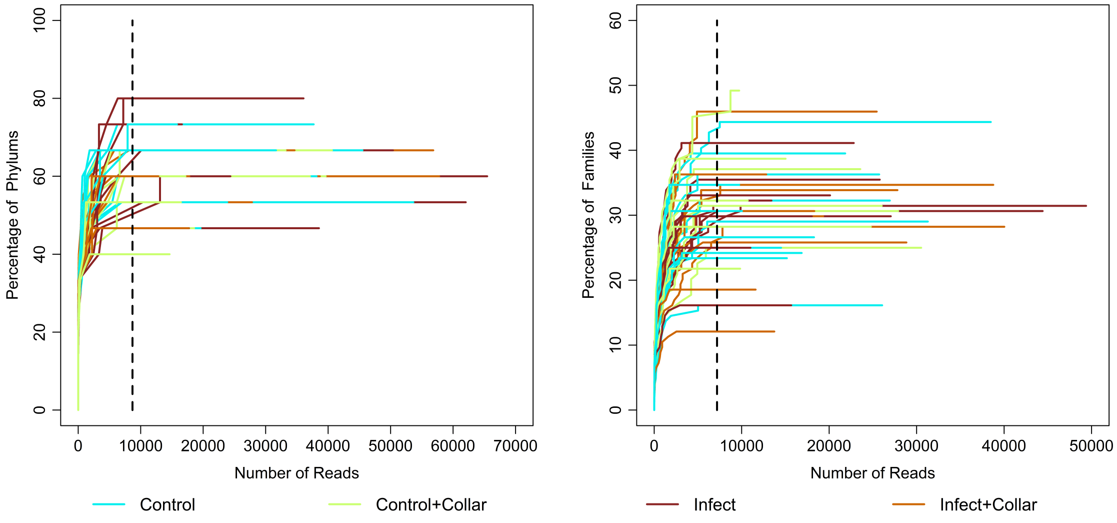

Table B. Bacterial diversity index and number of taxa by treatment (Infect, Infect+Collar, Control+Collar and Control) and sampling time at the 3 taxonomic levels. Only the significant ANOVA values (threshold: $p<0.05$) with the Sum of Squares (SS) and degree of freedom (df) from the linear regressions are reported.

Experimental level		*Shannon-Weaver* *SS, df, p*	*Simpson* *SS, df, p*	*Abundance* *SS, df, p*
Phylum	treatment			
	day			
	treatment*day			
Family	treatment	0.88, 3, 0.020		
	day			819.39, 2, 0.003
	treatment*day	1.34, 6, 0.023	0.06, 6, 0.053	895.02, 6, 0.044
Genus	treatment	2.64, 3, 0.001	0.13, 3, 0.001	1133.6, 3, 0.043
	day	0.81, 2, 0.024		1543.1, 2, 0.005
	treatment*day			

Figure B. PCoA plots of the microbiota by treatment (Infect: I, Infect+Collar: IC, Control+Collar: CC, and Control, C) at the 3 taxonomic levels (phylum, family and genus) using the Bray-Curtis diversity matrix. Circles represent the animals belonging to different treatments (colors). The variability explained by the two components (%), the ellipses clustering each treatment and the centers of the ellipses (or average value of every treatment, star) are reported. Treatments tend to overlap although there is a tendency for a strongest difference between Control+Collar and Infect cases.

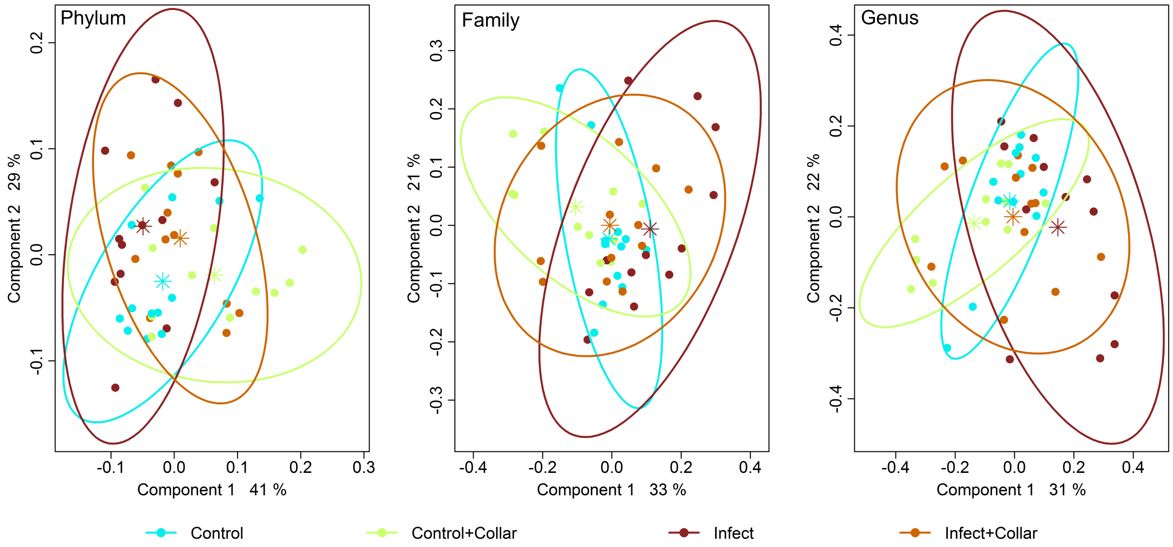

Table C. Variability in the abundance of the different bacterial taxa by treatment (Infect, Infect+Collar, Control+Collar and Control) and sampling time at the 3 taxonomic levels. Only the significant PerMANOVA values (1000 permutations, threshold: p<0.05) with the Sum of Squares (SS) and degree of freedom (df) using the Bray-Curtis diversity matrix are reported.

	Phylum<br>*SS, df, p*	*Family*<br>*SS, df, p*	*Genus*<br>*SS, df, p*
Treatment	0.93, 3, <0.01	1.46, 3, <0.001	1.53, 3, <0.001
Day	0.63, 2, <0.01	0.60, 2, <0.05	0.59, 2, <0.05
Treatment*Day	ns	ns	ns

Table D. Elliptical distances between the centers of ellipses depicted in the microbial diversity PCoA of figure S2. The largest distance is between Infect and Control+Collar treatments (bold values).

Treatment	*Phylum*	*Family*	*Genus*
C-CC	0.082	0.111	0.129
C-I	0.060	0.119	0.172
C-IC	0.049	0.023	0.036
CC-I	**0.122**	**0.218**	**0.282**
CC-IC	0.064	0.104	0.132
I-IC	0.060	0.116	0.153

Table E. Relationship between immune variables, as a response, and treatment and sampling time as independent variables. Only the significant ANOVA values (threshold: p<0.05) with the Sum of Squares (SS) and degree of freedom (df) from the linear regressions are reported.

Immune variables	*Treatment SS, df, p*	*Sampling time SS, df, p*	*Treat.*Sampl. time SS, df, p*
IFNγ	67.03, 3, <0.0001		
Tbet	3.86, 3, <0.05		
IL13	35.23, 3, <0.0001	7.38, 2, <0.05	
IL4	20.54, 3, <0.0001		
IL5			
GATA3			
IL10	24.96, 3, <0.0001		
FoxP3	3.60, 3, =0.0507		
RORγ	4.82, 3, <0.05		
TGFβ		2.13, 2, <0.05	
MUC2	56.58, 3, =0.0508		
MUC5AC			
IgA somatic	68.36, 3, <0.0001		13.03, 6, <0.05
IgA total	13.97, 3, <0.0001	4.31, 2, <0.01	4.49, 6, <0.05

Table F. *Post-hoc* pairwise multiple comparison Dunnett's Modified Tukey-Kramer test of the mean differences in the immune variables between treatments (Infect -I, Infect+Collar -IC, Control+Collar -CC and Control C). Treatment groups that do not share the same label are significantly different (threshold: $p<0.05$). Label rank is as follow: A>B>C. For example: for IFNγ, I and IC treatments have A in common, meaning IFNγ is similar between these two groups while C treatment has B, meaning IFNγ expression is higher in I than in C. Some treatments have two labels, e.g. A, B; that is, this treatment is similar to either label A or label B treatment. A treatment with label A and B is higher than a treatment with label C but it is not higher than a treatment with label B and C.

Immune variables	*I*	*IC*	*C*	*CC*
IFNγ	A	A	B	B
Tbet	A, B	A, B	B, C	C
IL13	A	A	B	B
IL4	A	A	B	B
IL5	A	A	A	A
GATA3	A	A	A	A
IL10	A	A	B	B
FoxP3	A	A	A	A
RORγ	B, C	A, B	A	A, B
TGFβ	A	A	A	A
MUC2	A	A	A	A
MUC5AC	A	A	A	A
IgA somatic	A	A	B	B
IgA total	A	A	B	B

Figure C. PCoA of the rabbit immune response (cytokine/transcription factor and function gene) by treatment (Infect: I, Infect+Collar: IC, Control+Collar: CC, and Control: C) using the Bray-Curtis diversity matrix. Circles represent the animals belonging to different treatments (colors). The variability explained by the two components, the ellipses clustering each treatment and the centers of the ellipses (or average value of every treatment, star) are reported. Infected animals group more closely together and cluster away from the group of the non-infected cases.

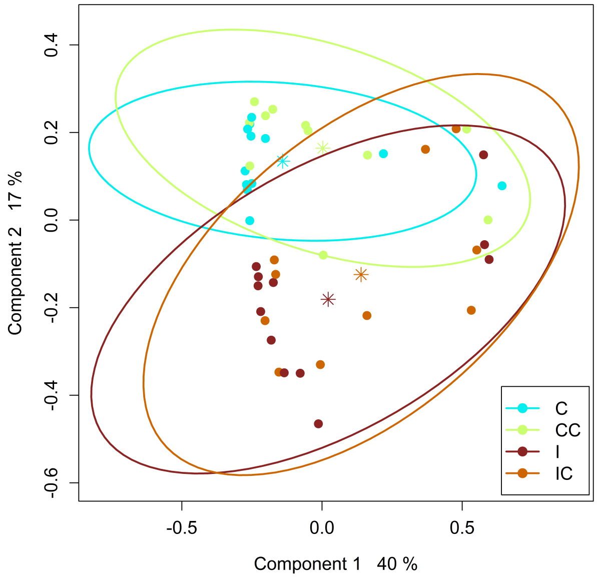

Table G. Elliptical distances between the centers of ellipses depicted in the immune response diversity PCoA of figure S3. The largest distance is between Infect+Collar and Control treatments (bold value).

Treatment	*Ellipse Distances*
C-CC	0.146
C-I	0.355
C-IC	**0.381**
CC-I	0.346
CC-IC	0.319
I-IC	0.130

Figure D. *T. retortaeformis* abundance (mean ± s.e., log-transformed), in the small intestine duodenum by treatment and time post initial infection. There is no significant difference in parasite abundance among treatments and sampling time (*post-hoc* Dunnett's Modified Tukey-Kramer test for all p>0.05).

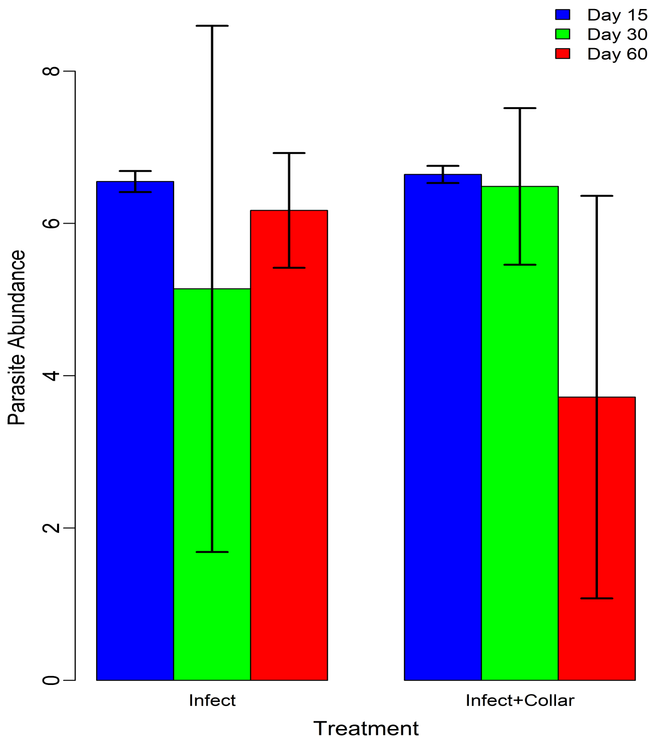

Figure E. PCoA of the duodenal microbiota functionality by treatment (Infect: I, Infect+Collar: IC, Control+Collar: CC, and Control: C) using the Bray-Curtis diversity matrix. Circles represent the animals belonging to different treatments (colors). The variability explained by the two components, the ellipses clustering each treatment and the centers of the ellipses (or average value of every treatment, star) are reported. All treatments tend to overlap. Bacterial functionality tends to be similar among treatments.

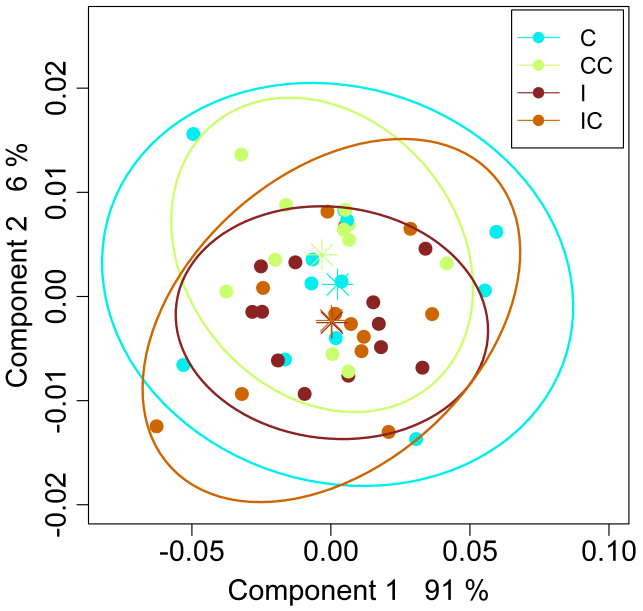

Table H. Elliptical distances between the centers of ellipses depicted in the microbiota functionality PCoA of figure S5. The largest distance is between Control+Collar and Infected treatments (bold value).

Treatment	*Ellipse Distances*
C-CC	0.00625
C-I	0.00421
C-IC	0.00413
CC-I	**0.00739**
CC-IC	0.00712
I-IC	0.00028

Figure F. Rabbit body mass (mean ± s.e.) by treatment (Infect: I, Infect+Collar: IC, Control+Collar: CC, and Control: C) and time post initial infection. Body mass significantly increases over time and differs among treatment and the interaction treatment-time (ANOVA of the linear model, for all: $p<0.0001$). *Post-hoc* pairwise multiple comparison Dunnett's Modified Tukey-Kramer test between treatments found that CC is significantly lower than C, I and IC; no significant differences were observed between the other treatments.

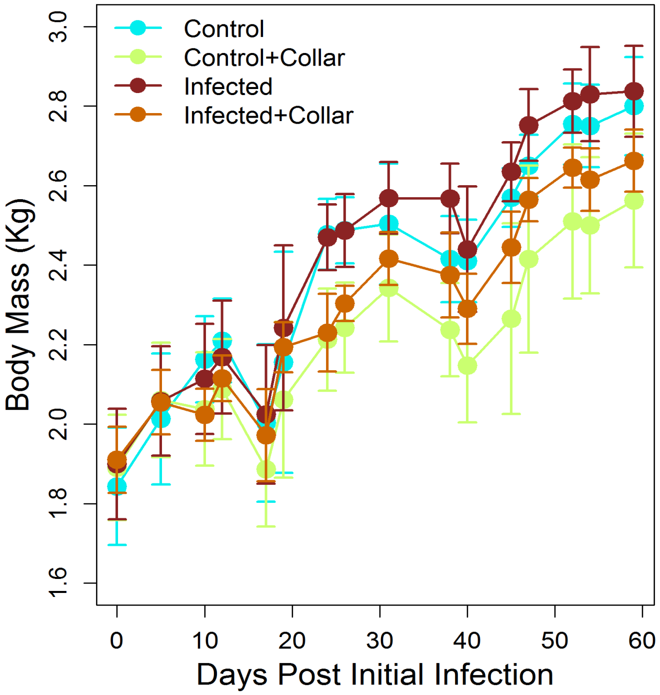

Supplement: S1 File — Text A. Quantification of total IgA antibodies. Table A. Primer and probe sequences for the immune variables. Table B. Bacterial diversity index and abundance by treatment. Table C. Variability in the abundance of the bacterial community by treatment and sampling time. Table D. Elliptical distances of the PCoA in Figure B in S1 File. Table E. Variability of every immune variable by treatment and sampling time. Table F. Post-hoc pairwise comparison of the immune variables between treatments. Table G. Elliptical distances of the PCoA in Figure C in S1 File. Table H. Elliptical distances of the PCoA in Figure E in S1 File. Figure A. Rarefaction Analysis. Figure B. PCoA plots of the microbiota by treatment. Figure C. PCoA of the rabbit immune response by treatment. Figure D. T. retortaeformis abundance by treatment and time post initial infection. Figure E. PCoA of the duodenal microbiota functionality by treatment. Figure F. Rabbit body mass by treatment and time post initial infection. (PDF) [file pone.0159770.s001.pdf]
